# Long-Term Patient-Reported Bowel and Urinary Quality of Life in Patients Treated with Intensity-Modulated Radiotherapy Versus Intensity-Modulated Proton Therapy for Localized Prostate Cancer

**DOI:** 10.3390/curroncol32040212

**Published:** 2025-04-02

**Authors:** Kimberly R. Gergelis, Miao Bai, Jiasen Ma, David M. Routman, Bradley J. Stish, Brian J. Davis, Thomas M. Pisansky, Thomas J. Whitaker, Richard Choo

**Affiliations:** 1 Department of Radiation Oncology, University of Rochester School of Medicine and Dentistry, 601 Elmwood Ave, Rochester, NY 14642, USA; 2 Department of Operations and Information Management, University of Connecticut, 352 Mansfield Rd, Storrs, CT 06269, USA; 3 Department of Radiation Oncology, Mayo Clinic, 200 First St SW, Rochester, MN 55905, USA; 4 Department of Radiation Physics, Division of Radiation Oncology, The University of Texas MD Anderson Cancer Center, 1515 Holcombe Blvd, Houston, TX 77030, USA

**Keywords:** quality of life, EPIC-26, prostate cancer, intensity-modulated radiotherapy, intensity-modulated proton therapy

## Abstract

Purpose: This study aimed to compare long-term patient-reported outcomes in bowel and urinary domains between intensity-modulated radiotherapy (IMRT) and intensity-modulated proton therapy (IMPT) for localized prostate cancer. Methods and Materials: Patients with clinical T1–T2 prostate cancer receiving IMRT or IMPT at a tertiary cancer center from 2015–2018 were analyzed to determine the changes in the prospectively collected bowel function (BF), urinary irritative/obstructive symptoms (UO), and urinary incontinence (UI) domains of EPIC-26. The mean changes in EPIC-26 scores were evaluated from pretreatment to 24 months post-radiotherapy for each modality. A score change >50% of the baseline standard deviation was considered a clinically meaningful change. Results: A total of 82 patients treated with IMRT (52.2%) and 56 patients treated with IMPT (53.3%) completed the questionnaire at baseline and 24 months post-RT. There were no baseline differences in domain scores between treatment modalities. At 24 months post-radiotherapy, there was a significant and clinically meaningful decline in the BF mean score in the IMRT cohort (−4.52 (range −50, 29.17), *p* = 0.003), whereas the decline in BF score did not reach clinical relevance or significance (−1.88 (range −37.5, 50), *p* = 0.046) when accounting for the Bonferroni adjustment in the IMPT cohort. A higher proportion of patients treated with IMRT had a clinically relevant reduction in BF when compared with IMPT (47.37% vs. 25.93%, *p* = 0.017). The mean changes in the UI and UO scores of the IMRT and IMPT cohorts were neither statically significant nor clinically relevant. Conclusions: IMPT leads to a smaller decrease in BF than IMRT at 24 months post-RT, while there was no differential effect on UO and UI.

## 1. Introduction

Treatment-related adverse effects and overall quality of life (QOL) are essential outcomes in prostate cancer treatment. Definitive prostate cancer treatments, including radical prostatectomy and radiotherapy (RT), can alter bladder, bowel, and sexual function [1]. Minimizing treatment-related adverse effects and maintaining QOL are crucial in patients’ treatment choices.

Intensity-modulated RT (IMRT) and proton beam therapy (PBT) are conformal radiotherapy modalities that allow for dose escalation to the prostate while minimizing the dose delivered to adjacent normal structures. PBT has been used as a means of potentially increasing the therapeutic ratio of external beam RT for prostate cancer. Protons can lower the dose of radiation delivered to at-risk organs due to its Bragg peak dose distribution profile, and it potentially reduces radiation toxicity, compared to photons [2]. The question of whether this dosimetric advantage translates to decreased toxicity is of great interest.

As several single-institution studies of PBT for prostate cancer yielded encouraging results, two phase III studies investigating PBT versus IMRT for the treatment of prostate cancer are underway (ClinicalTrials.gov identifiers: NCT01617161, NCT04083937) [3,4,5,6]. The primary endpoint of both the Prostate Advanced Radiation Technologies Investigating Quality of Life (PARTIQoL) and Prostate Cancer Patients Treated with Alternative Radiation Oncology Strategies (PAROS) trials is to determine whether PBT is superior to IMRT in patient-reported bowel QOL. The PARTIQoL group recently presented their results in abstract form; the results demonstrated no difference between PBT or IMRT in the mean change in bowel score at 24 mo (*p* = 0.836), with both arms showing only a small, clinically non-meaningful decline from the baseline [7]. Patients treated with PBT on PARTIQoL could have received either pencil-beam scanning intensity-modulated proton therapy (IMPT) (49%) or passive scatter (51%) PBT. Approximately half of included patients received hypofractionation (51%).

Our group previously reported the effect of IMRT versus IMPT for prostate cancer on early urinary and bowel toxicity using the prospectively collected 26-item Expanded Prostate Index Composite (EPIC-26) [5]. This study demonstrated the significant and clinically meaningful worsening of bowel function (BF) and urinary obstructive (UO) symptom scores at the end of RT for patients receiving either IMRT or IMPT. The IMRT cohort experienced greater decrease in BF scores and had a higher proportion of patients with clinically meaningful reductions compared to IMPT. The IMRT group had significant and clinically meaningful worsening of BF at three months post-RT, whereas the change in the BF score of the IMPT cohort was no longer statistically significant nor clinically meaningful compared to the baseline. Changes in UO or urinary incontinence (UI) were neither significant nor clinically meaningful three months post-RT.

Late toxicity and QOL changes are also meaningful endpoints of interest to patients and providers. Comparative-effectiveness studies of conventional RT, three-dimensional conformal RT (3DCRT), IMRT, and PBT have been reported; however, these studies relied on Medicare claims as surrogates for clinical outcomes [8,9,10,11,12] that used conventional fractionation or passively scattered PBT [13,14]. As moderate hypofractionation is now an attractive alternative [15] and IMPT is increasingly used, we herein report the late toxicity outcomes of IMRT and IMPT using prospectively collected EPIC-26 data from patients treated with conventionally fractionated or moderately hypofractionated RT.

## 2. Materials and Methods

### 2.1. Patient Selection

Our cohort was selected using an Institutional-Review-Board-approved prospective registry of patients who received IMRT or IMPT to the prostate ± the proximal seminal vesicles for clinical stage T1–T2 N0 M0 prostate cancer at a tertiary cancer center between 2015 and 2018. Patients receiving pelvic lymph node irradiation were excluded. The included patients completed the EPIC-26 questionnaire prior to the initiation of RT, as well as 24 ± 9 months post-RT. All included patients were treated with 60 Gy in 20 fractions, 70.2 Gy in 26 fractions, or 78 Gy in 39 fractions, as these regimens were the most commonly utilized at our institution during this period. Ultrahypofractionated regimens were excluded.

### 2.2. Measurement of Patient-Reported QOL and Treatment Details

Patients prospectively completed the EPIC-26 questionnaire prior to RT (baseline), at the completion of RT, and at 3, 6, 12, 24, 36, and 48 months post-RT. The EPIC-26 questionnaire includes domain scores for BF, UO, UI, sexual function (SF), and hormonal function (HF). Responses within each domain are scored from 0–100, with higher scores indicating better function [16].

The primary outcomes included the change in BF, UO, and UI scores from pretreatment to 24 months post-RT. SF and HF domains were excluded as they were confounded by the use of androgen deprivation therapy (ADT).

Details of IMPT and IMRT treatment planning at our institution have been previously described [5].

### 2.3. Statistical Analysis

Differences in the demographics, clinical features, and baseline EPIC-26 BF, UO, and UI scores between patients treated with IMRT and those receiving IMPT were initially examined to assess whether a meaningful analysis of QOL change between the two modalities could be performed. The 24-month time point was selected to investigate late toxicity as 24 ± 9 months was identified as having the greatest number of responders. Baseline characteristics between the responders and the non-responders to the 24-month questionnaire were compared to assess whether the outcomes obtained from the responders could be generalized to the overall patient population.

The changes in BF, UO, and UI scores from baseline to 24 months post-RT were compared between patients treated with IMRT and those receiving IMPT. The statistical significance and clinical relevance of the score changes at 24 months post-RT were also assessed for IMRT and IMPT. A clinically meaningful change was defined as a score change that exceeded >50% of the standard deviation of a baseline score [17,18,19]. The difference in the proportion of patients with clinically meaningful changes between treatment with IMRT and IMPT was also assessed.

We examined the mean changes in EPIC-26 scores from baseline to 24 months post-RT for each RT modality to evaluate the late effects of RT on QOL. The evaluation of EPIC-26 score changes was limited to patients who completed the questionnaire both at baseline and at 24 ± 9 months post-RT.

We evaluated the independent effects of multiple variables to determine factors that significantly impacted EPIC-26 score changes over time. The examined variables included the RT modality, dose-fractionation regimen, use of a rectal hydrogel spacer, baseline EPIC-26 scores for BF, UO, and UI domains, age, baseline prostate-specific antigen (PSA), Gleason score, T stage, and the receipt of ADT. Due to the small number of non-White patients, race was excluded from this analysis. Stepwise backward/forward variable selections were conducted for the model construction based on the Akaike Information Criterion (AIC). Only variables with statistical significance were tabulated.

A two-sided Wilcoxon rank–sum test, a Wilcoxon signed-rank sum test, Fisher’s exact test, and a *t*-test were used for the testing of statistical hypotheses, with *p* < 0.05 considered statistically significant unless specified otherwise. For analyses involving multiple pairwise comparisons, *p* < 0.017 was considered statistically significant to account for Bonferroni correction.

## 3. Results

### 3.1. Baseline Demographics and EPIC-26 Questionnaire Completion

A total of 157 patients treated with IMRT and 105 patients treated with IMPT completed the baseline pre-RT EPIC-26 questionnaire. Of those, 82 patients treated with IMRT (52.2%), and 56 patients treated with IMPT (53.3%) also completed the questionnaire at 24 months post-RT, and these patients comprised our study cohort. There were no statistical differences between the responders and non-responders with respect to age, baseline PSA, Gleason score, T stage, dose-fractionation regimen, the proportion of patients with hydrogel spacer (40% vs. 43%), the proportion of patients receiving androgen deprivation therapy (75% vs. 75%), baseline BF score (93.3 vs. 93.5), baseline UI score (88.2 vs. 87.7), or baseline UO score (85.3 vs. 87.6).

Table 1 describes the patient demographics and treatment characteristics of the IMRT and IMPT cohorts. There were no differences between the IMRT and IMPT cohorts with respect to race, mean age, mean pre-RT serum prostate-specific antigen (PSA), Gleason score, PSA distribution, or the proportion of patients receiving hydrogel spacer. IMPT patients were more likely to have T2 disease than those receiving IMRT (69.6% vs. 51.2%, *p* = 0.04). There was no difference in the proportion of patients receiving each dose-fractionation regimen between IMRT and IMPT (42.7% vs. 25.0% for 60 Gy in 20 fractions; 47.6% vs. 64.3% for 70.2 Gy in 26 fractions; 9.8% vs. 10.7% for 78 Gy in 39 fractions, respectively).

### 3.2. Changes in the BF, UO and UI Scores Between the IMRT and IMPT Cohorts

There were no statistically significant differences in the baseline BF, UO, and UI domain scores between the IMRT and IMPT cohorts (Table 1).

In the IMRT cohort, there was a clinically relevant and significant decline in the BF mean score from baseline to 24 months post-RT (*p* = 0.003) (Table 2). In contrast, the decline in the BF mean score in the IMPT cohort was less pronounced and did not reach statistical significance when accounting for the Bonferroni adjustment (*p* = 0.046). Furthermore, its decline was not a clinically relevant reduction. The mean changes in the UI and UO scores of the IMRT and IMPT cohorts were neither statistically significant nor clinically relevant.

A higher proportion of patients treated with IMRT had a clinically relevant reduction in BF when compared with IMPT (47.4% vs. 25.9%, *p* = 0.017), which approached significance. There was no difference in the proportion of patients with clinically relevant reductions in UI (*p* = 0.82) or UO (*p* = 0.36) scores between IMRT and IMPT (Table 3).

The mean score changes in BF, UO, and UI domains from baseline to 24 months post-RT of the IMRT and IMPT cohorts are described and compared in Table 4. The mean BF and UI scores declined in both cohorts. The mean score of the UO domain improved from baseline in the IMPT cohort, whereas it declined in the IMRT cohort. Although the mean score decline in the BF domain was less in the IMPT cohort (−1.88) than in the IMRT cohort (−4.52), the difference in the mean score decline between the two cohorts was not statistically significant. There were also no statistical differences in the mean score changes in UI and UO domains when comparing the IMRT and IMPT cohorts.

### 3.3. Variables Associated with Domain Score Changes

The variables associated with the domain score changes from baseline to 24 months post-RT are summarized in Table 5. A greater reduction in the BF domain for all patients was associated with a higher baseline BF score (i.e., fewer bowel symptoms) (*p* < 0.001) and a lower baseline UO score (i.e., more irritative/obstructive symptoms) (*p* = 0.01). A greater reduction in the UO domain was associated with a higher baseline UO score (i.e., fewer irritative/obstructive symptoms) (*p* ≤ 0.001). A greater reduction in the UI domain was associated with a higher baseline UI score (i.e., less urinary incontinence) (*p* < 0.001). The domain score changes were not associated with the radiation modality, dose-fractionation regimens, or use of a hydrogel spacer.

## 4. Discussion

Our group previously reported early toxicity differences between IMRT and IMPT prostate RT, showing that the IMRT cohort experienced greater decrease in BF at the end of RT, and that a higher proportion of patients have a clinically meaningful reduction compared with IMPT. The IMRT group exhibited a significant and clinically meaningful worsening of BF at three months following the completion of RT, whereas the change in BF score of the IMPT cohort was neither statistically significant nor clinically meaningful compared to the baseline. At three months following the completion of RT, there were no statistically significant nor clinically meaningful changes in UO or UI. As early toxicity generally improves with time, an evaluation of late effects is an important addition to the toxicity profile of any treatment, which prompted this study.

With continued follow up, we found that patients treated with IMRT had a significant and clinically meaningful decrease in BF two years after completing treatment, whereas IMPT- treated patients did not. In addition, the percentage of patients with a clinically relevant reduction in BF score was greater with IMRT than with IMPT, and this approached significance. However, in our multivariable linear regression analysis, we did not identify an association between the treatment modality and a change in the BF domain score from baseline to 24 months post-RT. Only better baseline BF and worse baseline UO scores were associated with decline in BF score at 24 months. This lack of an association between the treatment modality and changes in the BF domain score according to the multivariate analysis is possibly due to the small number of patients included in this study, with response rates at 24 months of 52.2% and 53.3% for IMRT and IMPT, respectively. That is, our sample size was insufficient to detect a difference, if one existed. There were no significant or clinically meaningful differences in UI or UO domain scores at 24 months between patients treated with IMRT or IMPT.

Hoppe et al. completed a similar study at the University of Florida [13] and, similarly, found that only BF scores met the minimally detectable difference from baseline at six months, one year, and two years for IMRT, and at one year and two years for PBT. In their study, more patients treated with IMRT had a minimally detectable difference in BF score from baseline at six months (*p* = 0.002); however, there was no difference between modalities at one and two years. This differs from our study, because the patients treated with IMRT at our institution reported a clinically meaningful reduction in BF at two years compared to patients treated with IMPT. However, our studies have differences that may affect the outcomes and interpretation of results. Hoppe et al. compared a group treated with conventionally fractionated (1.8 Gy or 2.0 Gy per fraction) passive scatter PBT at their center with IMRT patients treated at nine other centers during an earlier timeframe. In contrast, our patients mainly received moderately hypofractionated (2.7 Gy or 3.0 Gy per fraction) RT at only one institution. Our report may be more germane to contemporary practice, because hypofractionated RT has become a standard of care for organ-confined prostate carcinoma. The patients in our study were treated with pencil-beam IMPT, rather than passive scatter, which is a newer and more conformal technology.

A matched-pair analysis conducted by Dutz et al. compared patient-reported early and late toxicities using the EORTC-QLQ-C30/PR25, dosimetric parameters, and QOL between conventionally fractionated passive scatter PBT and IMRT in prostate cancer patients [20]. Their study identified significantly lower late urinary urgency in patients receiving PBT, and it also found that late grade ≥ 2 GU toxicities were associated with the relative volume of the anterior bladder wall receiving 70 Gy and the entire bladder receiving 60 Gy. Their study is limited by the use of passive scatter and patient-reported QOL only for one year.

The University of Washington reported their experience [21] of patient-reported QOL with conventionally fractioned IMPT for prostate cancer and found that EPIC bowel domain declined at one-year post-treatment compared to baseline with no further decline over time; however, the IMPT cohort was not compared to an IMRT cohort. They also had a more heterogeneous study cohort than ours, as some of their patients also received pelvic nodal RT. Pugh et al. conducted a similar study [22] investigating the QOL of patients receiving conventionally fractionated passive scatter PBT or IMPT for prostate cancer; similarly, they found a clinically significant decrement in bowel QOL over time. However, there was no IMRT comparative cohort in their study.

Bulman et al. investigated the rectal dose and changes in bowel-related QOL in patients treated with IMPT and correlated QOL to dosimetric parameters [23]. Their study examined a heterogeneous cohort, including patients treated with conventional, moderate hypofractionation, as well as ultrahypofractionation. The BF domain score declined from baseline to the end of treatment and at 12 months; however, this study did not investigate whether it was a clinically meaningful decline. They found that rectal BED D25 (Gy) ≥23% was significantly associated with decline.

The Phase III PARTIQoL study recently presented results in abstract form; these results demonstrated no difference between PBT or IMRT in the mean change in the bowel score at 24 months (*p* = 0.836), with both arms showing only small, clinically non-meaningful decline from baseline [6,7]. This study included a large number of patients treated with PBT and IMRT (n = 226 and n = 224, respectively). Like our cohort, participants received treatment to the prostate and proximal seminal vesicles; pelvic nodal RT was not performed. Our results differ from those of the PARTIQoL study; in our cohort, patients treated with IMRT had a significant and clinically meaningful decrement in BF two years after completing treatment, whereas the IMPT- treated patients did not. Both IMPT (49%) and passive scatter (51%) techniques were utilized in the PBT cohort of PARTIQoL, whereas all patients in our PBT cohort received IMPT. It is possible that the PBT technique or bowel and/or rectum dosimetry may account for this discrepancy. It is also possible that we detected differences in bowel QOL by chance.

Our study has multiple limitations. First, our study was not designed as a randomized clinical trial. Thus, it does not offer a definitive comparison between IMRT and IMPT, and there may be selection bias and unknown factors affecting treatment outcomes and the type of treatment modality a patient received. Second, our evaluation of QOL change was limited to a single post-RT timepoint, because not every patient answered the EPIC-26 surveys at each specified time point. Treatment-related QOL may change over time, and we were not able to capture this information or to present the QOL data as a continuum. Third, we had a relatively small number of patients, and this may introduce some uncertainty that a larger study cohort would not face. Thus, the results of our study need to be interpreted with caution. Fourth, we did not examine whether a potential benefit of IMPT in BF outcome justifies the additional cost of proton therapy. Lastly, although our study suggests that patients treated with IMPT experience lower rates of late BF decline, we did not correlate this with the dose-volume histogram (DVH) parameters used in planning RT.

## 5. Conclusions

Most previously published reports of patients treated with IMRT and PBT for localized prostate cancer have found a decline in BF QOL after RT. Our study provides a single institution’s comparative analysis between IMRT and IMPT for BF, UO, and UI changes at 24 months post-RT using prospectively collected EPIC-26 data. In our experience, IMPT continued to show less of a decrease in BF than IMRT at 24 months post-RT, whereas there was no differential effect on UO and UI between treatment modalities. Further work, including dosimetric studies correlating QOL with DVH parameters and randomized trials, are needed to determine whether there is a clinically meaningful difference in tumor control, RT adverse effects, and QOL changes between IMRT and IMPT.

## Figures and Tables

**Table 1:** Baseline characteristics of the IMRT cohort vs. the IMPT cohort.

Characteristics	IMRT (n = 82)	IMPT (n = 56)	*p*-Value *
Age			
Mean (range), year	70.9 (55–84)	70.7 (52–88)	0.77
Age group, n (%)			0.95
<70	33 (40.2%)	21 (37.5%)	
70–79	46 (56.1%)	33 (58.9%)	
≥80	3 (3.7%)	2 (3.6%)	
Race			
Race, n (%)			1.00
White	77 (93.9%)	53 (94.6%)	
Others	3 (3.7%)	2 (3.6%)	
Missing	2 (2.4%)	1 (1.8%)	
Pre-RT PSA			
Mean (range, ng/mL)	7.8 (0.2–39.4)	7.5 (0.2–23.2)	0.64
PSA level, n (%)			0.30
<4	25 (30.5%)	11 (19.6%)	
4–10	40 (48.8%)	34 (60.7%)	
>10	17 (20.7%)	11 (19.6%)	
Gleason score			
Group, n (%)			0.63
≤7	68 (82.9%)	49 (87.5%)	
>7	14 (17.1%)	7 (12.5%)	
T stage			
Group, n (%)			0.04
T1	40 (48.8%)	17 (30.4%)	
T2	42 (51.2%)	39 (69.6%)	
Dose-fractionation regimen			
n (%)			0.10
60 Gy/20 fractions	35 (42.7%)	14 (25.0%)	
70.2 Gy/26 fractions	39 (47.6%)	36 (64.3%)	
78 Gy/39 fractions	8 (9.8%)	6 (10.7%)	
Hydrogel spacer			
Treated with hydrogel spacer, n (%)	35 (42.7%)	20 (35.7%)	0.48
Androgen deprivation therapy (ADT)			
Treated with ADT, n (%)	64 (78.1%)	40 (71.4%)	0.42
Baseline bowel score			
Mean (range)	94.0 (62.5–100)	92.3 (50–100)	0.98
Standard deviation	8.3	12.6	
Missing	3	1	
Baseline urinary incontinence score			
Mean (range)	87.7 (22.8–99.5)	88.9 (39.3–99.5)	0.49
Standard deviation	16.7	15.8	
Missing	0	1	
Baseline urinary irritative/obstructive score			
Mean (range)	85.8 (43.8–100)	84.4 (50–100)	0.50
Standard deviation	12.6	12.6	
Missing	0	5	

* *p*-values were derived from Wilcoxon rank–sum test for continuous variables, and Fisher’s exact test for categorical variables. *p*-values reflect whether the means or the distributions were different between the two cohorts. *p* < 0.05 is considered significant. IMRT: Intensity Modulated Radiation Therapy. IMPT: Intensity Modulated Proton Therapy. Pre-RT PSA: Pre-radiotherapy prostate-specific antigen. T stage: Tumor stage. Gy: Gray. ADT: Androgen deprivation therapy

**Table 2:** Score changes and their clinical relevance for each treatment modality.

EPIC-26 Domain	Radiation Modality	No. of Respondents (%)	Mean Score Change From Baseline (Range)	*p* *	Clinically Relevant Change? (Y/N) **
Bowel function	IMRT	76 (92.7%)	−4.52 (−50, 29.17)	0.003	Y
	IMPT	54 (96.4%)	−1.88 (−37.5, 50)	0.046	N
Urinary incontinence	IMRT	67 (81.7%)	−2.61 (−32.75, 27.5)	0.79	N
	IMPT	51 (91.1%)	−0.80 (−39, 40)	0.75	N
Urinary irritative/obstructive	IMRT	76 (92.7%)	−0.58 (−50, 25)	0.76	N
	IMPT	51 (91.1%)	1.84 (−37.5, 43.75)	0.41	N

* *p*-values were derived from a Wilcoxon signed-rank–sum test and reflect whether late scores were different from baseline score. *p* < 0.017 is considered significant to account for the Bonferroni adjustment. ** When a score change exceeds > 50% of the standard deviation of baseline score, it is considered clinically relevant. IMPT: Intensity Modulated Proton Therapy. IMRT: Intensity Modulated Radiation Therapy. EPIC-26: 26-item Expanded Prostate Index Composite. Y: Yes. N: No.

**Table 3:** Comparison of the proportions of patients with clinically relevant changes between the IMRT cohort and the IMPT cohort.

EPIC-26 Domain	Radiation Modality	Patients with Clinically Relevant Reduction (%)	*p* *
Bowel			
	IMRT	36 (47.4%)	0.017
	IMPT	14 (25.9%)	
Urinary Incontinence			
	IMRT	15 (22.4%)	0.82
	IMPT	10 (19.6%)	
Urinary Irritative/Obstructive			
	IMRT	12 (15.8%)	0.36
	IMPT	12 (23.5%)	

* *p*-values derived from Fisher’s exact test. *p*-values test whether the difference in the proportion of patients experiencing clinically relevant reduction is statistically significant between two modalities. *p* < 0.017 is considered significant to account for the Bonferroni adjustment. IMPT: Intensity Modulated Proton Therapy. IMRT: Intensity Modulated Radiation Therapy. EPIC-26: 26-item Expanded Prostate Index Composite

**Table 4:** Comparison of score changes between the IMRT cohort and IMPT cohort.

EPIC-26 Domain	Radiation Modality	Late Response	
		Mean Change from Baseline	*p* *
Bowel			
	IMRT	−4.52	0.69
	IMPT	−1.88	
Urinary Incontinence			
	IMRT	−2.61	0.55
	IMPT	−0.80	
Urinary Irritative/Obstructive			
	IMRT	−0.58	0.64
	IMPT	1.84	

* *p*-values were derived from a Wilcoxon rank–sum test. *p*-values reflect whether score changes were different between two treatment cohorts. *p* < 0.017 is considered significant to account for the Bonferroni correction. IMPT: Intensity Modulated Proton Therapy IMRT: Intensity Modulated Radiation Therapy EPIC-26: 26-item Expanded Prostate Index Composite

**Table 5:** Factors associated with late changes in each domain of the EPIC-26 score.

EPIC-26 Domain	Independent Variable	Coefficient	*p*-Value *
Bowel	Baseline BF score	−0.9	<0.001
	Baseline UO score	0.2	0.01
Urinary incontinence	Baseline UI score	−0.4	<0.001
Urinary irritative/obstructive	Baseline UO score	−0.7	<0.001

* Multivariable linear regression was used to identify factors that were statistically significantly associated with changes in the scores. *p*-values test the individual covariate effect. *p* < 0.05 is considered significant. Variables included in the regression models were the type of radiation modality (IMPT vs. IMRT), the dose-fractionation regimen utilized (60 Gy/20 fraction vs. 70.2 Gy/26 fraction vs. 78 Gy/39 fraction), the use of a hydrogel spacer (yes vs. no), baseline BF, UO, and UI scores, age, PSA, the Gleason score, the T stage, and the use of ADT. We excluded race from this analysis because of the small number of non-Caucasian patients. Stepwise backward/forward variable selections were conducted for the model construction based on the Akaike Information Criterion (AIC). Only those variables found to be statistically significant are tabulated. EPIC-26: 26-item Expanded Prostate Index Composite. BF: Bowel function symptom score. UO: Urinary irritative/obstructive symptom score. UI: Urinary incontinence symptom score

## Data Availability

The data presented in this study are available on request from the corresponding author due to institutional requirements regarding data sharing.
